# Order-by-disorder from bond-dependent exchange and intensity signature of nodal quasiparticles in a honeycomb cobaltate

**DOI:** 10.1038/s41467-021-23851-0

**Published:** 2021-06-24

**Authors:** M. Elliot, P. A. McClarty, D. Prabhakaran, R. D. Johnson, H. C. Walker, P. Manuel, R. Coldea

**Affiliations:** 1 Clarendon Laboratory, University of Oxford, Oxford, UK; 2 Max Planck Institute for the Physics of Complex Systems, Dresden, Germany; 3 Department of Physics and Astronomy, University College London, London, UK; 4 ISIS Facility, Rutherford Appleton Laboratory-STFC, Didcot, UK

**Keywords:** Magnetic properties and materials, Phase transitions and critical phenomena

## Abstract

Recent theoretical proposals have argued that cobaltates with edge-sharing octahedral coordination can have significant bond-dependent exchange couplings thus offering a platform in 3*d* ions for such physics beyond the much-explored realisations in 4*d* and 5*d* materials. Here we present high-resolution inelastic neutron scattering data within the magnetically ordered phase of the stacked honeycomb magnet CoTiO_3_ revealing the presence of a finite energy gap and demonstrate that this implies the presence of bond-dependent anisotropic couplings. We also show through an extensive theoretical analysis that the gap further implies the existence of a quantum order-by-disorder mechanism that, in this material, crucially involves virtual crystal field fluctuations. Our data also provide an experimental observation of a universal winding of the scattering intensity in angular scans around linear band-touching points for both magnons and dispersive spin-orbit excitons, which is directly related to the non-trivial topology of the quasiparticle wavefunction in momentum space near nodal points.

## Abstract (web summary)

It was suggested that some 3d materials display bond-dependent exchange interactions, leading to exotic many-body effects. Here, using inelastic neutron scattering, the authors reveal such interactions in the stacked honeycomb magnet CoTiO_3_ and show how they induce a spectral gap and affect the Dirac magnon band structure.

## Introduction

Spin-orbit coupling is at the origin of many remarkable properties of condensed matter uncovered in recent years^1–5^. It is central to the appearance of nontrivial topological invariants in electronic band structures and underlies the existence of bond-dependent exchange couplings that have been shown to bring about exotic features in many quantum magnets^6–8^. In the latter case, much of the effort in materials discovery has focussed on heavy 5*d* and 4*d* ions in which the spin-orbit coupling is one of the dominant energy scales. Notable are the honeycomb iridates *A*_2_IrO_3_ (*A* = Na, Li) and related materials, and *α*-RuCl_3_, which displayed a range of many novel exotic magnetic properties including spin-momentum locking^9^, incommensurate orders with counter-rotating spin spirals^6^, broad scattering continua in the spectrum of spin excitations^10^ or unconventional field-dependent thermal Hall effect^11^. The origin of these exotic forms of behaviour is the presence of significant anisotropic, bond-dependent exchange, which in extreme cases has been predicted to stabilise quantum spin liquids, such as the celebrated Kitaev honeycomb model with Ising exchanges along orthogonal directions for the three bonds that meet at each site^12^. The path to the discovery of the unusual magnetic properties of those materials has been a fruitful one starting with theoretical proposals that bond-dependent exchange couplings can arise in certain iridates and ruthenates with edge-sharing octahedra^13,14^. The octahedra supply a crystal field environment that leads to an effective low-energy spin one-half degree of freedom for the magnetic ions and the edge-sharing provides the local exchange pathway that, in conjunction with the spin-orbit coupling, produces anisotropic bond-dependent exchange. Now there is evidence for significant such exchanges in honeycomb iridates and ruthenates^6–8^.

More recent theoretical work has argued that significant bond-dependent exchange in the form of Kitaev and related couplings may also arise between Co^2+^ ions in edge-sharing octahedral coordination^15–17^ thus extending the original proposals into a surprising new setting. To investigate such effects we report here inelastic neutron scattering (INS) measurements of the spin dynamics in the stacked honeycomb magnet CoTiO_3_. Our data show propagating spin-wave excitations with a clear low energy spectral gap, which was inferred but could not be resolved by previous studies^18^. We show that the spin-wave spectrum is not merely compatible with the presence of bond-dependent exchange, but that such couplings must be present in the low energy pseudo-spin one-half theory in order to explain the origin of the gap. Moreover, we show that the gap opening must occur via a quantum order-by-disorder mechanism^19–25^ as a consequence of unusually strong constraints on the possible mechanisms that can open the spectral gap. In view of the low-lying crystal field excitations in this material compared to the exchange coupling, we provide compelling evidence that virtual crystal field excitations are the driving mechanism for order-by-disorder^26,27^ assisted by spin-orbital exchange and supply a calculation of the spin-wave spectrum including this effect that captures the principal features of the data.

CoTiO_3_ is part of a growing list of materials^18,28,29^ explored as candidates displaying Dirac magnons. Earlier studies established the presence of Dirac nodal lines^18^, which make this material ideal for the exploration of a recently predicted^30^ fingerprint of a topologically non-trivial magnon band structure, namely a universal azimuthal modulation in the dynamical structure factor around linear band touching points, not probed experimentally before and which originates from the special topological features in the wavefunction of nodal quasiparticles. We indeed observe clear evidence for the predicted intensity winding around the nodal points, thus providing a direct measurement of the non-trivial topology of the Dirac magnon wavefunctions and establishing that there are meaningful features in the momentum-and-energy dependent dynamical structure factor beyond simply revealing the quasiparticle dispersion relations. Furthermore, we observe analogous features in the dispersive spin-orbital excitations at higher energy, highlighting the universal properties of Dirac bosonic quasiparticles. Finally, we investigate the effect of the bond-dependent exchange on the Dirac nodal lines arguing that they are robust to gap opening and likely appear as ‘double helices’ winding around each zone corner. We show that the same type of bond-dependent anisotropic exchange that opens up the spectral gap provides a natural explanation for a ‘double-peak’ structure in energy scans near the nodal points.

## Results

### Magnon dispersions

**Fig. 1:**
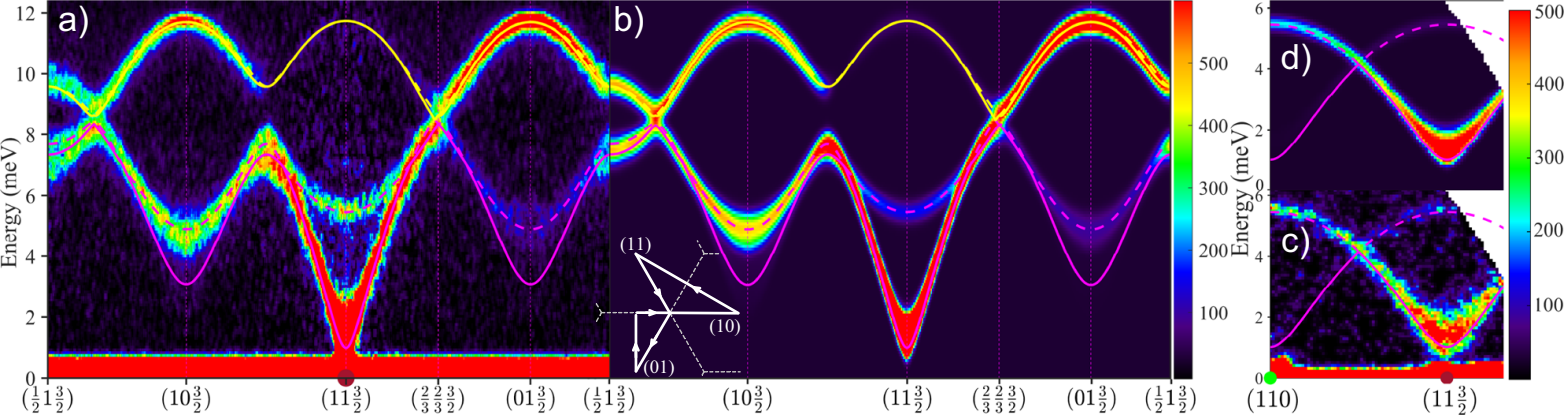
Magnon dispersions. INS data at 8 K observing the magnon dispersions along high-symmetry directions **a** in-plane and **c** out-of-plane, compared in **b** and **d** with the XXZΔ model. Lines are the model dispersions $\tilde{\omega }({\bf{k}})$, green/brown dots on the elastic line indicate location of structural/magnetic Bragg peaks. Lower left inset in **b** shows the wavevector path in **a**, **b** (arrowed solid white lines) projected onto the (*hk*) plane, grey dashed lines are the 2D Brillouin zone boundaries. Intensities are averaged for a transverse wavevector range of ±0.1 Å^−1^. The incident neutron energy was *E*_i_ = 18 meV in **a** and 9.6 meV in **c**. The colour bars indicate scattering intensity in arbitrary units on a linear scale.

The magnon dispersions along high-symmetry directions in the honeycomb plane obtained using INS measurements on single crystals of CoTiO_3_ (for details see Supplementary Note 6A) are summarised in Fig. 1a. Wavevectors are indexed in reciprocal lattice units of the hexagonal structural unit cell. Near the (1, 1, 3/2) magnetic Bragg peak the lowest mode has a near-linear in-plane dispersion. As the honeycomb layers are ferromagnetically ordered with moments confined to the crystallographic *ab* plane, the linear dispersion indicates predominant easy-plane-type exchange couplings for in-plane neighbours. Figure 1c observes a finite dispersion at low energies in the direction normal to the layers, indicating finite inter-layer couplings, and a small but finite spectral gap Δ = 1.0(1) meV, clearly resolved above the magnetic Bragg peak. Reference^18^ proposed that a finite spin gap would be needed to account for the observed non-linear magnetisation curve in small in-plane fields^31^, but it was not possible to directly resolve the gap excitation in the earlier lower-resolution INS data^18^. Apart from the finite gap, the main features of the magnon spectrum can be accounted for by a minimal exchange Hamiltonian ${{\mathcal{H}}}_{{\rm{XXZ}}}$ for the stacked honeycomb geometry in CoTiO_3_, allowing for each bond a different exchange coupling between the moment components along the *c*-axis, and between the components in the *ab* plane. For a single ferromagnetic honeycomb layer, two magnon bands (acoustic/optic) would be expected with linear crossings at the corners (K-points) of the hexagonal Brillouin zone. For finite interlayer couplings that stabilise antiferromagnetic stacking of layers, the number of bands doubles and inter-layer resolved lower bands are expected with almost degenerate higher bands, as observed in Fig. 1a, c. ${{\mathcal{H}}}_{{\rm{XXZ}}}$ has a gapless (Goldstone) mode corresponding to moments rotating freely in the *ab* plane, so to capture the observed gap we assume that the physical mechanism responsible for gap generation only modifies the dispersion relations *ω*(**k**) of ${{\mathcal{H}}}_{{\rm{XXZ}}}$ by adding a gap in quadrature, i.e. experimental dispersion points are compared with $\tilde{\omega }({\bf{k}})=\sqrt{{\omega }^{2}({\bf{k}})+{{{\Delta }}}^{2}}$. We call this parameterisation the XXZΔ model to emphasise that the gap Δ is not intrinsic, but is an additional, empirical fitting parameter. We find that exchanges up to 6th nearest-neighbour (nn) are important and obtain a very good level of agreement for both the dispersions and intensities as shown by comparing Fig. 1a with b, and c with d (for more details see Supplementary Notes 5B, C and 6C).

### Quantum order-by-disorder

The presence of the finite magnon spectral gap Δ is important as it indicates preferential moment orientations inside the easy plane. The magnetic ground state of Co^2+^ (3*d*^7^) ions in the local crystal field environment is a Kramers doublet with pseudospin-1/2, for which there is no local anisotropy, so any preferential orientation must be selected by interactions beyond the minimal ${{\mathcal{H}}}_{{\rm{XXZ}}}$ Hamiltonian. We focus our attention on bilinear couplings in the pseudospin as higher-order two-site couplings project down to such couplings. As outlined in Supplementary Note 10, multi-site couplings will be suppressed by the large charge gap. As there is no detectable distortion of the crystal lattice following the onset of the magnetic order, we perform the analysis of bi-linear couplings between cobalt moments that are symmetry-allowed by the crystal structure space group. We find that whilst various bond-dependent exchange couplings can be present in principle, at the classical level, surprisingly, the ground state energy remains independent of the moment orientation in the *ab* plane—see Supplementary Notes 7 and 8.

This degeneracy must however be an artefact of the mean-field approximation, as the real material Hamiltonian has only discrete, rather than continuous rotational symmetry around the *c*-axis. Such degeneracies would, in general, be expected to be lifted by quantum fluctuations via an order-by-disorder mechanism^19–24^, when the ground state energy (per site) acquires a contribution from zero-point fluctuations of the form ${\epsilon }_{{\rm{qu}}}(\phi)=\frac{1}{2}{\sum }_{m}\langle {\omega }_{m}({\bf{k}})\rangle$, where *ϕ* defines the moments’ orientation in the *ab*-plane relative to the *a*-axis and 〈*ω*_*m*_(**k**)〉 is the average energy of dispersive branch *m* = 1–4 over the Brillouin zone. The possibility that an order-by-disorder mechanism might be relevant for the ground state selection in CoTiO_3_ was mentioned in^18^, but no quantitative model was proposed. We show by direct calculations in Supplementary Note 8 that the semi-classical degeneracy is indeed lifted by zero-point fluctuations from bond-dependent anisotropic couplings such as *η* ≡ *J*^*yy*^ − *J*^*xx*^ on the 1st neighbour bond where *y* defines the local bond direction and *x* is in-plane transverse to *y*, and we find an induced gap that scales as Δ ~ ∣*η*∣^3/2^ at leading order. At the level of the low energy pseudospin-1/2 moments this provides a natural qualitative mechanism for the observed gap. One can also place this finding in the context of a theory that operates within the full set of 12 single-ion spin and orbital states. In fact, working within the pseudospin-1/2 picture suggests an unphysically large coupling *η* calculated in Supplementary Note 8 compared to the coupling *η* fitted in Supplementary Note 7. Since the crystal field excitations are comparable to the exchange scale, an entirely natural mechanism for order-by-disorder to arise is through virtual crystal field fluctuations in a model that includes small spin–orbital exchange. The virtual crystal field mechanism has been discussed in the context of Er_2_Ti_2_O_7_^26,27^—essentially the only other well-characterised example of order-by-disorder—where the linear spin-wave mechanism and virtual crystal field mechanism are complementary. However, in CoTiO_3_ virtual crystal field excitations are the leading cause of the discrete symmetry breaking. A so-called flavour-wave expansion^32–36^ incorporating this effect captures the magnon dispersions including the spectral gap and the dispersing crystal field excitations, as shown in Supplementary Note 10.

### Neutron intensity fingerprint of magnon isospin winding

**Fig. 2:**
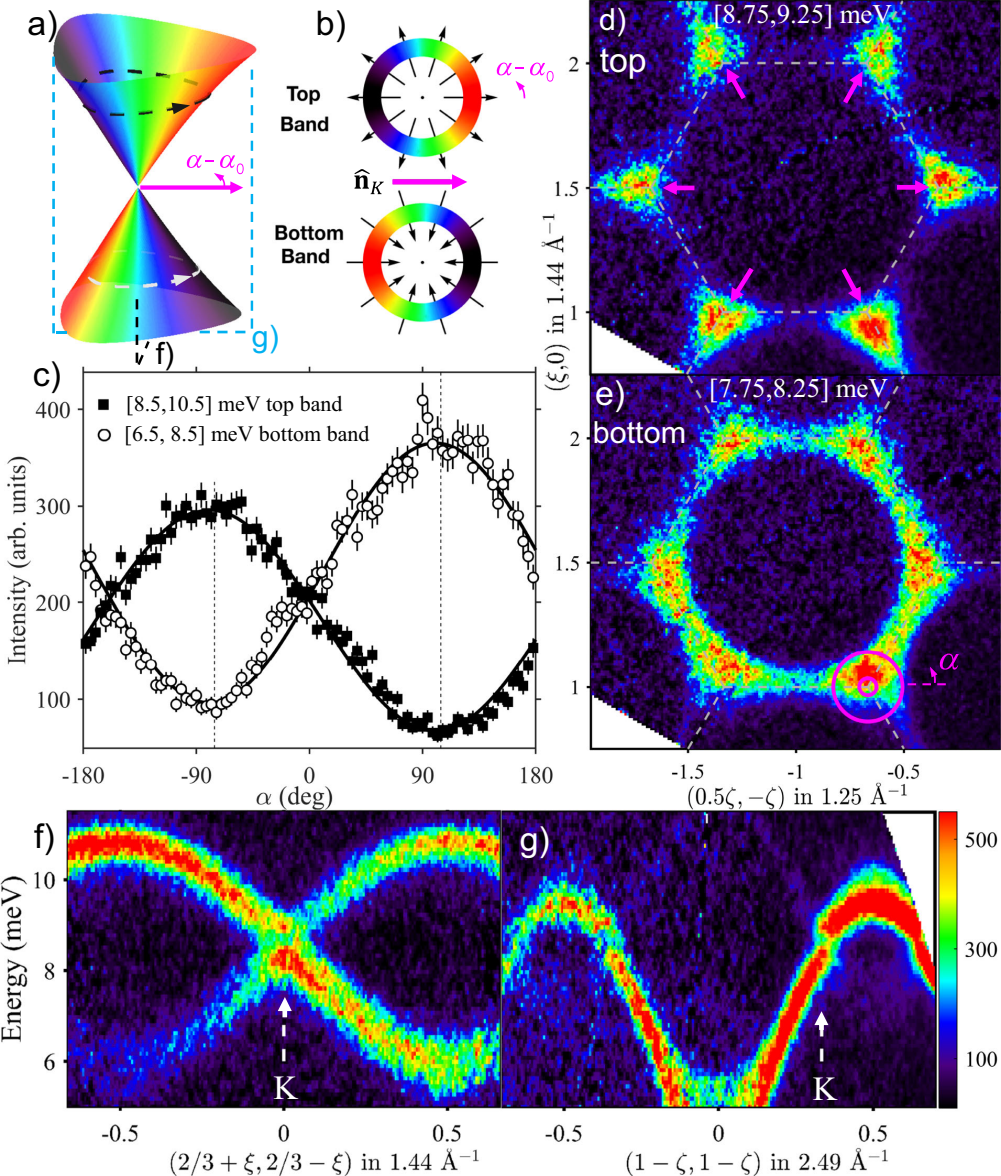
Intensity winding for Dirac magnons: theory and experiment. **a** Conical dispersion surfaces meeting at a Dirac node for a honeycomb ferromagnet. **b** Winding pattern of the isospin polarization $\boldsymbol{\sigma}$ (radial arrows) out/in from the nodal point for the top/bottom bands. In both **a**, **b** colour is the dynamical structure factor $1+{\boldsymbol{\sigma }}\cdot {\hat{{\bf{n}}}}_{{\rm{K}}}$, which winds around the node in antiphase between the top and bottom bands. **c** Constant-energy INS intensity in CoTiO_3_ as a function of azimuthal angle $\alpha$ around the (2/3,2/3) Dirac node, showing expected two-fold winding periodicity in anti-phase between the top/bottom bands (filled/open symbols) in agreement with **b**. The black squares/white circles denote the inelastic neutron scattering intensity for the top/bottom bands, with error bars representing one standard deviation. Solid lines are fits to cosine dependencies described in the text with dotted vertical lines showing the extreme points 180^∘^ apart. **d**/**e** Momentum intensity maps above/below the Dirac node energy, highlighting dramatic changes in the angular intensity dependence around the Dirac nodes. Dashed grey lines outline the edges of the 2D Brillouin zones and radial magenta arrows in **d** indicate the direction of the vectors $\hat{{\bf{n}}}$ at the zone corners at *L* = 0. Magenta annular region in **e** shows the radial in-plane wavevector range [0.05, 0.2] Å^−1^ in the angular scans in **c**. **f**/**g** INS data through a nodal point (vertical dashed arrow) along orthogonal in-plane directions that maximise the intensity asymmetry effect (slices shown in **a**) by dashed black/cyan rectangles, respectively): in **f** both crossing modes are visible, in **g** only one mode carries weight. All data were collected with *E*_i_ = 18 meV. In **c**–**g** intensities are averaged for *L* = [0, 2.4], and in **f** and **g** for an in-plane transverse momentum range of ±0.026 and ±0.028 Å^−1^, respectively. The colour bar in **g** applies also to **d**–**f**, indicating scattering intensity in arbitrary units on a linear scale.

Having established the presence of bond-dependent exchange in this material, we now focus on the Dirac points in the magnon spectrum which provide an ideal setting to explore predicted intensity modulations associated with the isospin winding around nodal points. To explain this physics we use the simple example of a two-dimensional (2D) honeycomb Heisenberg ferromagnet ${\mathcal{H}}=-J{\sum }_{\langle i,j\rangle }{{\bf{S}}}_{i}\cdot {{\bf{S}}}_{j}$ (*J* > 0) taken from ref. ^30^ to which we refer for further details, generalisations to band structures in 3D, and different types of touching points. The magnon band structure for this model computed within linear spin-wave theory around the collinear ferromagnetic ground state has Dirac points at finite frequency at the corners (K-points) of the 2D hexagonal Brillouin zone (dashed outline in Fig. 2d). For a small momentum *δ***k** measured from a Dirac node, the effective spin-wave Hamiltonian takes the famous form ${{\mathcal{H}}}_{{\rm{eff}}}=v\,\delta {\bf{k}}\cdot {\boldsymbol{\sigma }}$ where $v$ = 3*JSa*_0_/2 is the Dirac velocity (*a*_0_ is the nn distance) and the isospin encoded in the Pauli matrices $\boldsymbol{\sigma}$ originates from the two sublattice honeycomb structure. By analogy with the Zeeman Hamiltonian, it follows that magnon wavefunctions carry an isospin polarisation that is locked to the offset momentum *δ***k** thus winding around each Dirac point, see Fig. 2b. This feature is directly observable via INS because, in the vicinity of these points, the intensity is, up to a constant, the projection of the isospin polarization onto some direction $\hat{{\bf{n}}}$ characteristic of each Dirac point^30^, illustrated by the pink radial arrows in Fig. 2d. Explicitly, the intensity takes the form $1\pm \cos (\alpha -{\alpha }_{0})$ where $\alpha$ is the polar angle around the K point and ${\alpha }_{0}$ defines the direction of $\hat{{\bf{n}}}$, with the upper/lower sign for the top/bottom band, respectively. Therefore, the intensity winds smoothly around the Dirac point as illustrated by the colour shading on the two conical bands in Fig. 2a.

### Isospin of Dirac magnons in CoTiO_3_

CoTiO_3_ provides a nearly ideal experimental platform to see the theoretically predicted winding of neutron intensity in the vicinity of the Dirac points. Figure 2f shows the INS data along the (1, −1) in-plane direction through the nominal Dirac point at (2/3,2/3) where a clear near-linear band crossing is observed. In contrast, Fig. 2g shows that the INS data through the same K point, but along the orthogonal (1,1) direction, has vanishingly small intensity in one of the two crossing bands. This strong intensity asymmetry in orthogonal scans is precisely what is expected based on the predicted isospin winding around a Dirac node in Fig. 2a. This can be seen more directly in Fig. 2c, which plots the intensity dependence as a function of angle $\alpha$ winding around the Dirac node in the top/bottom bands (filled/open symbols), the maxima and minima in each band are 180° apart and in anti-phase between the two bands, the solid lines show fits the generic form ${A}_{\pm }\pm {B}_{\pm }\cos (\alpha -{\alpha }_{0})$ with the upper/lower sign for top/bottom band. The fits give ${\alpha }_{0}$ = −80(3)°, in good agreement with the XXZΔ model for the same scan − 81(1)°, the offset from − 60° is due to the buckling of the honeycomb layers, which rotate the $\hat{{\bf{n}}}$ vectors in plane upon varying *L*, for more details see Supplementary Note 5. The observed two-fold angular dependence is precisely the fingerprint of the predicted isospin winding for the near-nodal quasiparticles.

### Fine structure of Dirac magnons

**Fig. 3:**
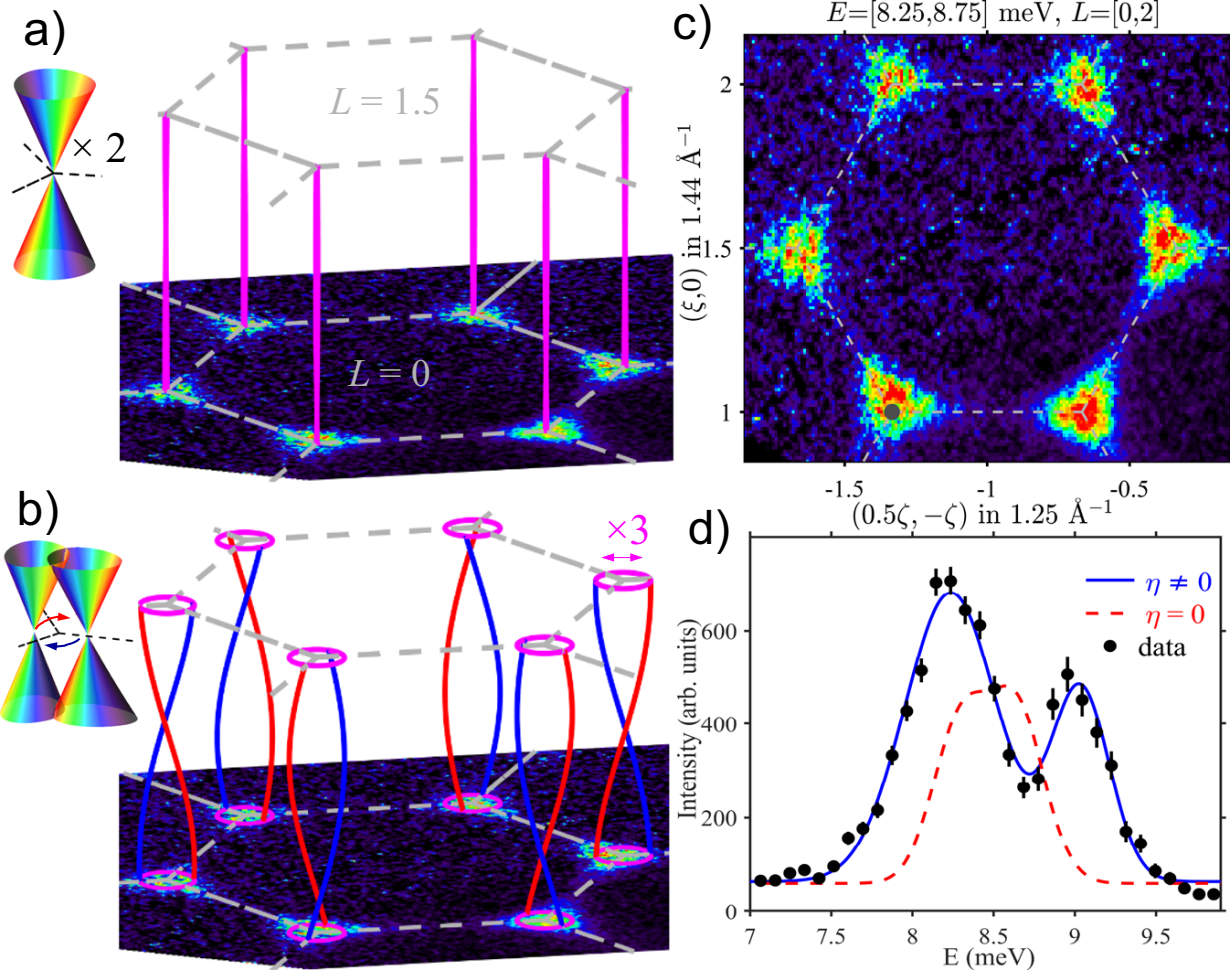
Dirac magnon nodal lines. Nodal lines along *L* for **a** Heisenberg and **b** XXZ interlayer couplings (blue/red lines correspond to in-/out-of-plane polarization). Top left insets show the band structure near the nodal points, two doubly-degenerate touching cones in **a** and momentum-offset pairs of touching cones in **b**, curly arrows indicate precession of the nodal points along *L*. In **b** the diameter of the 'double helix' nodal lines is amplified for visibility by ×3 compared to the XXZΔ model. **c** Momentum INS intensity map as in Fig. 2d, e, but centred at the nominal nodal energy. Dashed lines show 2D Brillouin zone edges. The intensities are on a linear scale as per the colour bar in Fig. 2g. **d** Energy scan averaged between all six K-points in **c** as well as (1/3,1/3), for a cylindrical wavevector range of in-plane radius 0.03 Å^−1^ (dark grey dot at (1/3,4/3) in **c** bottom left) and *L* = [0, 2.3]. Error bars represent one standard deviation, the dashed line is the calculated lineshape for the XXZΔ model (*η* = 0), and the solid line is a fit that includes an additional exchange anisotropy *η* = − 1.7 meV, both calculations include instrumental resolution effects.

The bond-dependent exchange that is responsible for the spectral gap also affects the Dirac nodal lines. For antiferromagnetic Heisenberg interlayer couplings, the nodal points form lines along *L*, each 4-fold degenerate (the top and bottom cones in Fig. 2a are each doubly degenerate due to the antiferromagnetic doubling of the number of magnetic sublattices). For an XXZ Hamiltonian two cases can occur depending on the anisotropy of the interlayer coupling *J*_2_: (i) for Heisenberg *J*_2_ the nodal lines are degenerate and are straight along *L* [see Fig. 3a], (ii) for XXZ *J*_2_ they are separated in momentum, but remain at the same energy and wind along *L* in a ‘double-helix’ [see Fig. 3b], in opposite senses between adjacent K-type points due to the $\bar{3}$ point group symmetry of the crystal lattice. However, neither of those cases can explain the fine structure observed by the energy scan in Fig. 3d centred at K-points, where two peaks are clearly resolved, 0.75(5) meV apart, instead of a single peak (XXZΔ model, dashed red line, case (ii) above; for case (i) the single peak would be even sharper). This fine structure was not detected by earlier lower-resolution studies^18^ and accounting for it requires anisotropic coupling terms beyond ${{\mathcal{H}}}_{{\rm{XXZ}}}$. We have already argued that such terms must be present in order to account for the spectral gap. As shown in Supplementary Note 9, these terms all preserve the Dirac nodal lines while shifting their position in momentum space along in-plane directions related to the moment orientation in the ground state. To make quantitative contact with the experiment, we demonstrate that adding a finite nearest neighbour bond-dependent exchange *η* leaves the dispersions largely unaffected relative to the *η* = 0 case in the magnetic Brillouin zone interior while leading to the observed double-peak structure in Fig. 3d (solid line).

### Dirac excitons

**Fig. 4:**
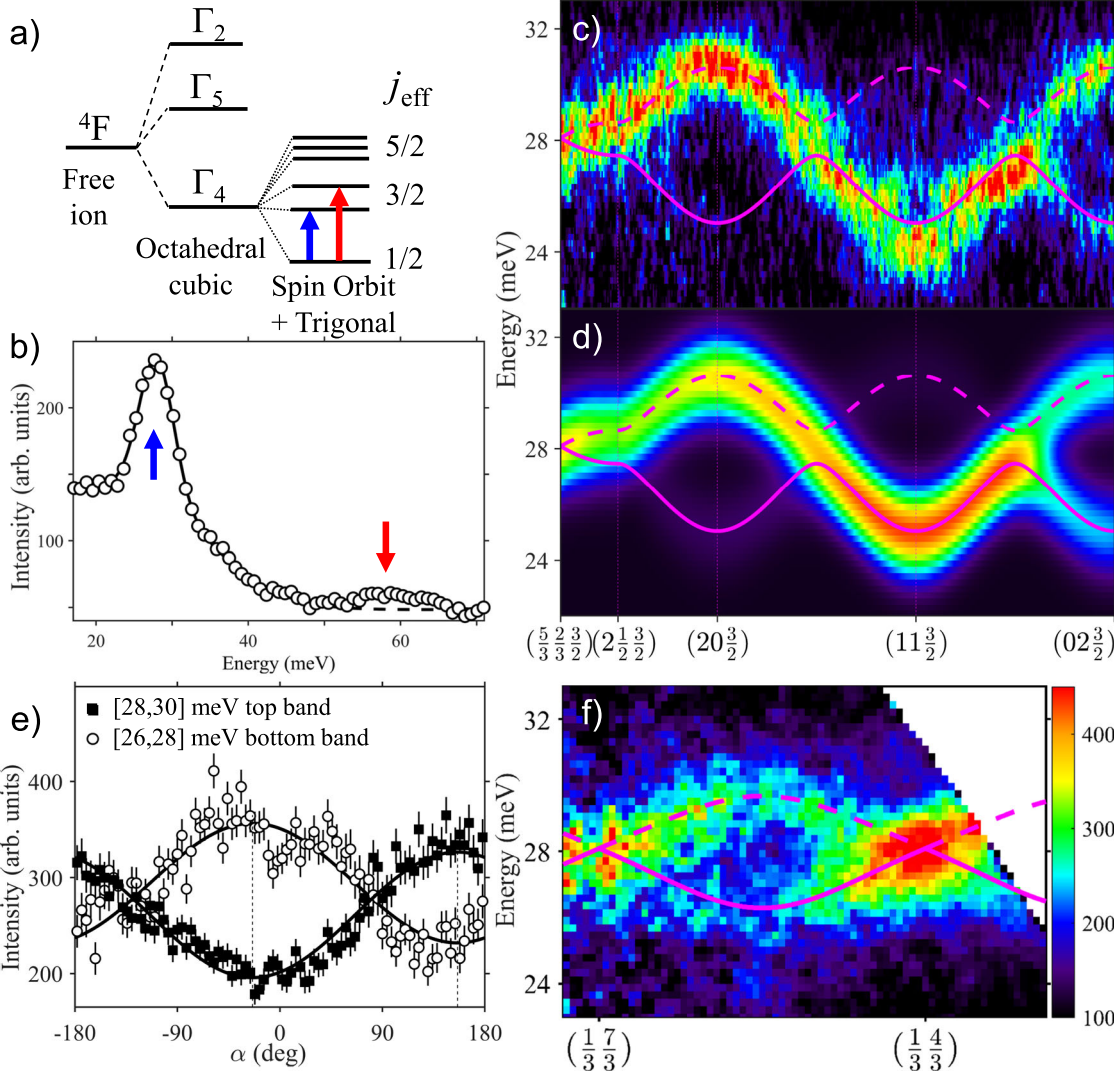
Spin-orbit excitons: dispersions and Dirac node. **a** Schematic level splitting for a Co^2+^ ion in an octahedral crystal field of trigonal symmetry including spin-orbit coupling. **b** INS energy scan observing transitions to the first two excited crystal field levels (the blue/red arrows above the peaks show the transitions indicated by matching colour vertical arrows in **a**, the solid line is a guide to the eye. **c** INS data probing the dispersions of the first crystal field level along high-symmetry directions, compared in **d** with a tight-binding model (thick solid/dashed lines through both graphs show best fit dispersions). **e** Angular intensity dependence around the nodal point (2/3,5/3) for the top/bottom exciton bands fitted to an ${{\mathcal{A}}}_{\pm }\pm {{\mathcal{B}}}_{\pm }\cos (\alpha -{\tilde{\alpha }}_{0})$ form (solid lines, ${\tilde{\alpha }}_{0}=155{(3)}^{\circ }$, calculated 153(1)^∘^, in-plane radial wavevector range [0.075, 0.3] Å^−1^). The black squares/white circles denote the inelastic neutron scattering intensity for the top/bottom exciton bands, respectively, with error bars representing one standard deviation. Note the analogous behaviour to the intensity dependence in azimuthal scans for Dirac magnons in Fig. 2c. **f** Exciton bands crossing at the two labelled nodal Dirac points, analogous to the magnon bands crossing in Fig. 2f). In **e**, **f** intensities are averaged for *L* = [0, 3.5], in **c** for a transverse wavevector range ±0.1 Å^−1^, and in **f** for a transverse in-plane wavevector range ±0.025 Å^−1^. Data were collected at 8 K with *E*_i_ = 83 meV in **b** and 45 meV in **c**, **e**, **f**. The colour bar in **f** also applies to **c** and **d**, and indicate scattering intensity in arbitrary units on a linear scale.

We now describe high-energy excitations, which we attribute to transitions to higher crystal field levels, where we also observe propagating excitations with linear band touching points and intensity winding around nodal points. The local spin-orbit coupled and trigonally distorted octahedral crystal field scheme for a Co^2+^ (3*d*^7^) ion (*L* = 3 and *S* = 3/2) is shown in Fig. 4a. Figure 4b shows INS measurements observing two peaks centred near 28 and 58 meV, which we identify with the (exciton) transitions to the two trigonally-split doublets of the *j*_eff_ = 3/2 excited quadruplet (blue and red vertical thick arrow in Fig. 4a).

Figure 4c shows higher resolution INS measurements observing clear in-plane dispersions for the lower exciton modes near 28 meV, attributed to hopping due to spin and orbital exchange. Two modes are expected due to the two sublattices of the honeycomb structure and Fig. 4f shows clear evidence for mode crossing at the two labelled nodal positions. Angular intensity maps around a nodal point in Fig. 4e show a clear two-fold angular dependence, in anti-phase between the top/bottom bands (filled/open symbols), as expected from the intensity winding picture, again in complete analogy with the spectroscopic signature seen for the Dirac magnon wavefunctions in Fig. 2c. The observed dispersions and relative intensities of the two exciton modes can be well captured by a tight-binding model, detailed in Supplementary Note 4. The experimental and modelled exciton dispersions are compared in Fig. 4c, d. We note that after this work was completed, ref. ^37^ appeared, also reporting INS measurements of the exciton dispersion in CoTiO_3_.

## Discussion

To summarise, we have reported INS measurements of the magnon dispersions in the stacked honeycomb CoTiO_3_, which reveal the presence of a spectral gap and Dirac nodal lines. We have shown that the gap implies the presence of significant bond-dependent anisotropic exchange originating from spin-orbit coupling and we have proposed a minimal model compatible with the experimental data to explain the discrete symmetry breaking via a quantum order-by-disorder mechanism. We have also observed key signatures of proximity to Dirac magnon physics through near-linear band touching and characteristic two-fold intensity periodicity in azimuthal scans attributed to the isospin winding around the Dirac node. The similar features seen also at the nodal band crossing in the spin-orbit excitons show that neutron scattering provides a window into the universal properties of highly constrained wavefunctions around linear band-touching points in bosonic systems in the solid state.

## Supplementary information

Supplementary Information

## Data Availability

The experimental data in this study is available from ref. ^38^.
